# London Panel Committee

**Published:** 1915-03-27

**Authors:** 


### LONDON PANEL COMMITTEE.

### PAYMENT OF PRACTITIONERS.

At a meeting of the London Panel Committee on March 23 it was agreed, in view of adjustments of lists rendered necessary by the enlistment of many insured persons, to accept a proposal by the Insurance Committee to pay practitioners for the second quarter of 1915 at the reduced rate of 9d. instead of 1s. for each half quarter. The Panel Committee, however, declined to approve of such a method of payment "until further order," but decided instead to ask the Insurance Committee if increased advances could not be made in the third and fourth quarters of the year, when, presumably, the necessary adjustments of the lists would have been made.

### Approved Societies and Certification.

Resolutions were passed calling the attention of the Commissioners to the action of approved societies in unreasonably refusing to pay insured persons beyond the date of the last certificate submitted, in insisting upon the production of weekly certificates in cases of prolonged incapacity for work, and in requiring panel practitioners to supply weekly certificates in respect of insured persons attending as out-patients at public institutions. It was also decided to invite representatives of approved societies to a conference on difficulties with regard to certification.

### Surcharging: A Difficulty.

In view of the ruling of the Commissioners in an appeal by a practitioner against a decision of the Sheffield Insurance Committee to the effect that a practitioner, before being surcharged for excessive prescribing, has the right of being heard by the whole Panel Committee and not merely by the Pharmacy Sub-Committee, the Committee agreed to suspend all action in so far as it had recommended the Insurance Committee to surcharge and to ask that Committee to postpone cases now before it, until practitioners shall have been given an opportunity of exercising their right of appearing before the full Panel Committee.

### Size of Practitioners' Lists.

The Panel Service Sub-Committee presented a tabulated statement showing that at the end of the medical year 1913, 60 per cent. of the practitioners on the panel had less than 1,000 insured persons on their lists, and 87 per cent. had less than 2,000 persons. Owing to the fact that a number of practitioners practising in partnership accepted insured persons in the name of the partnership instead of in their individual names, such partnerships had had to be regarded for the purposes of the analysis as single doctor's practices, and the majority of the 176 lists containing over 2,000 persons were actually the lists of practitioners practising in partnership.

### The Drug Tariff.

The Committee decided to accept an invitation by the Insurance Act Committee (Drug Traffic Sub-Committee) of the British Medical Association to appoint two representatives to assist in preparing a memorandum for submission to the Departmental Committee, but at the same time to prepare its own memorandum for submission to the same body. The Committee passed a resolution supporting the view of the British Medical Association that at present support should not be given to the establishment of a central bureau for checking chemists' accounts.

